# Autonomous Underwater Navigation and Optical Mapping in Unknown Natural Environments

**DOI:** 10.3390/s16081174

**Published:** 2016-07-26

**Authors:** Juan David Hernández, Klemen Istenič, Nuno Gracias, Narcís Palomeras, Ricard Campos, Eduard Vidal, Rafael García, Marc Carreras

**Affiliations:** Underwater Vision and Robotics Research Center (CIRS), Computer Vision and Robotics Institute (VICOROB), University of Girona, C\Pic de Peguera, 13 (La Creueta), 17003 Girona, Spain; ngracias@eia.udg.edu (N.G.); npalomer@eia.udg.edu (N.P.); rcampos@eia.udg.edu (R.C.); eduard.vidalgarcia@udg.edu (E.V.); rafael.garcia@udg.edu (R.G.); marc.carreras@udg.edu (M.C.)

**Keywords:** underwater, path planning, mapping, 3D reconstruction, ecology

## Abstract

We present an approach for navigating in unknown environments while, simultaneously, gathering information for inspecting underwater structures using an autonomous underwater vehicle (AUV). To accomplish this, we first use our pipeline for mapping and planning collision-free paths online, which endows an AUV with the capability to autonomously acquire optical data in close proximity. With that information, we then propose a reconstruction pipeline to create a photo-realistic textured 3D model of the inspected area. These 3D models are also of particular interest to other fields of study in marine sciences, since they can serve as base maps for environmental monitoring, thus allowing change detection of biological communities and their environment over time. Finally, we evaluate our approach using the Sparus II, a torpedo-shaped AUV, conducting inspection missions in a challenging, real-world and natural scenario.

## 1. Introduction

Environmental science is an interdisciplinary field that gathers together different natural sciences to study and determine the interactions of physical, chemical and biological components of the environment, as well as their effects on the organisms which inhabit it. An important objective in such studies is to establish a baseline that permits detecting changes and correlating them with possible underlying factors. In order to correctly identify such changes, it is necessary to conduct long-term and high-frequency observations of the studied ecosystem. To this end, and especially during the last decades, robotic systems have started being used to systematically collect such environmental data [1].

Marine scientists were among the first to capitalize on the use of robotic vehicles for environmental monitoring. Oceanographers, for instance, started using unmanned underwater vehicles (UUVs) to study deep marine environments and the seafloor [2]. However, although the majority of such early applications were devoted to monitoring marine habitats, nowadays there are a significant and increasing number of robots that contribute to other environmental science domains. The interested reader is encouraged to look into [1] for an extensive review of such contributions.

UUVs, are divided into two categories: remotely operated vehicles (ROVs), which need to be controlled by a human operator, and autonomous underwater vehicles (AUVs) that conduct (autonomously) a pre-established mission. While the former group has as a main drawback its dependence on a surface vessel to operate the vehicle, the second, on the other hand, involves important research challenges around localization, perception, mapping, path planning, and safety—just to mention a few. Therefore, an important part of the work in underwater environmental robotics research, especially that involving AUV, has concentrated on developing the basic functional modules that allow autonomous operation.

In their simplest form, robotic monitoring applications in underwater environments involve an AUV that follows a sequence of pre-calculated waypoints in order to collect data, which are retrieved after concluding the mission. In other words, the AUV behaves as a mobile sensor that explores and measures aspects of interest in an underwater environment. For this reason, another important body of research has been dedicated to developing pipelines that automatically and systematically process large amounts of information.

With the increased selection of sensors now included in the AUV allowing performance of underwater explorations, underwater 3D mapping now relies on acoustic multibeam [3,4] or sidescan sonars [5] to produce elevations maps. This ability to accurately map underwater environments yields high added value to any survey, as such results convey immense information easily interpretable by humans [6].

However, while these maps are indispensable for providing a rough approximation of the terrain, they are not able to sense more complex structures (e.g., they cannot represent concavities). For this reason, optical imaging is used to recover high quality 3D representation of small areas of interest in high resolution [7]. For an extensive review of various methods for underwater 3D reconstruction, the interested reader is referred to [8].

Furthermore, as a result of numerous readily-available off-the-shelf underwater camera systems, as well as custom-made systems for deep-sea explorations, an increasing number of biologists, geologists and archaeologists rely on optical imagery to survey marine benthic habitats [9,10,11,12], study hydrothermal vents and spreading ridges [13,14] as well as ancient shipwrecks and settlements [6,15,16]. Underwater imagery has also been used to identify and classify different benthic elements in the surveyed area [17,18,19] as well as to detect changes in the environment [20,21,22].

Whatever the type of information required to study an underwater environment (e.g., thermal, chemical, acoustic, optic, etc.), most of the surveys done with AUVs are conducted in a previously explored area so that the vehicle can navigate at a constant and safe altitude from the seafloor. In a typical application, the vehicle uses its on-board sensors to gather (environmental) data that is used to build thematic maps. However, recent and potential new applications require the AUV to navigate in close proximity to underwater structures and the seafloor. An example is the imaging and inspection of different structures such as underwater boulders [23] or confined natural spaces (e.g., underwater caves) [24]. In some of these cases, preliminary information about the structure to be inspected, such as its location and shape, permits determining the region of interest in advance, so that a coverage path is pre-calculated. The information obtained during the mission is used to correct or adjust the path to the goal online in order to adapt to the real shape of underwater structures [23]. Nonetheless, there are applications in which no previous information is available on the environment, or cannot be obtained autonomously. In such cases, preliminary work has focused on gathering data to characterize such environments [24], while relying on human supervision to ensure vehicle safety.

On that basis, the purpose of this paper is to propose a framework that endows an AUV with the capability to autonomously inspect environments for which no previous information is available. The framework consists of two main functional pipelines: (1) one that computes collision-free paths while simultaneously mapping the surroundings incrementally; (2) another that allows the reconstruction of various 3D representations (i.e., sparse, dense, meshed, textured) of the surveyed area using images gathered by an arbitrary camera setup during the mission. Due to the aforementioned constraints of underwater optical mapping, the latter pipeline establishes a range of distances at which the AUV must navigate, which represents a path constraint to be considered by the former functional pipeline. The resulting 3D reconstructions will serve as base maps for environmental monitoring of interest areas, allowing the detection of any change in biological communities and their environment on a temporal scale, and enabling a new way to visualize the evolution of wide areas in that temporal scale.

**Figure 1:**
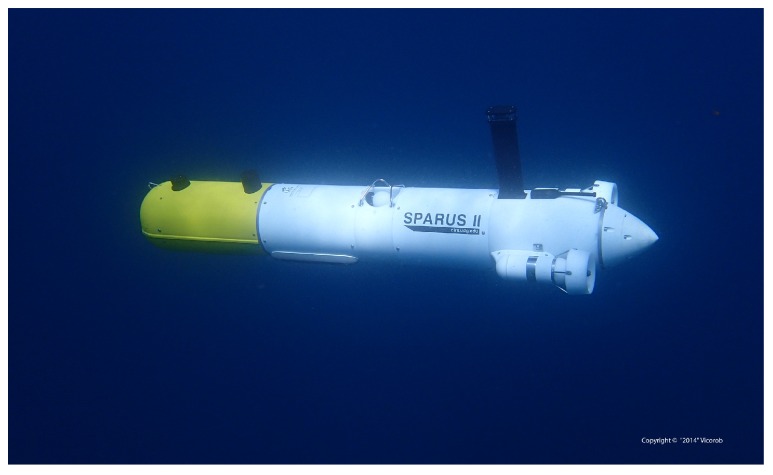
Sparus II, a torpedo-shaped AUV.

The remainder of this paper is organized as follows. Section 2 presents our proposed path planning pipeline that permits an AUV to autonomously navigate in unknown environments, and also discusses the extensions necessary in order to calculate paths that attempt to maintain a desired range of visibility, i.e., distance to inspected structure. Section 3 reviews the reconstruction pipeline that builds a 3D textured model of the inspected area using optical imagery. In Section 4, we present a real-world mission that validates our approach. Results include both an autonomous inspection conducted by the Sparus II (University of Girona, Girona, Spain) AUV (see Figure 1) in a challenging and natural environment, and its corresponding 3D reconstruction done with optical data gathered during the inspection mission. This clearly extends our preliminary work, where only simulated or real-world but structured (non-natural) environments have been used [25,26]. Finally, concluding remarks and directions for further research are given in Section 5.

## 2. Path Planning Pipeline

**Figure 2:**
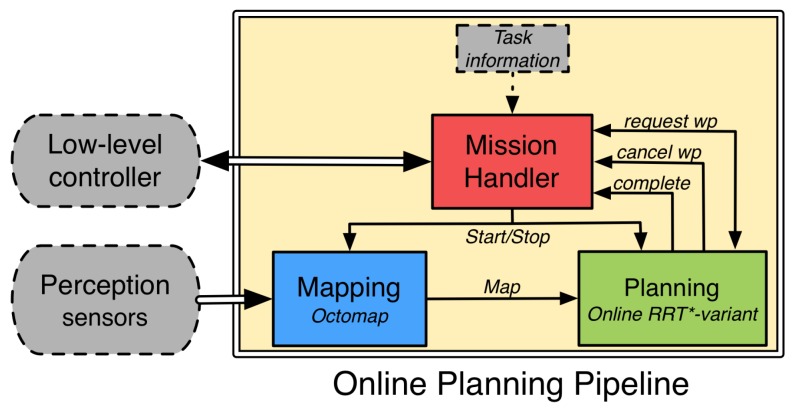
Pipeline for online path planning for AUV.

This section reviews our path-planning pipeline that solves start-to-goal queries online for an AUV that operates in unknown environments [25]. In order to accomplish this, the pipeline is composed of three functional modules. The first of them incrementally builds an occupancy *map* of the environment using on-board perception sensors. The second one *plans* safe (collision-free) paths online. The third and last functional module works as a high-level coordinator that *handles the mission* execution by exchanging information with the other two modules and the AUV’s controllers. Figure 2 depicts how these functional modules are connected to one another. Additionally, we explain how to extend this pipeline by incorporating a criterion to maintain a desired distance to guarantee visibility constraints while conducting a mission in close proximity.

### 2.1. Module for Incremental and Online Mapping

**Figure 3:**
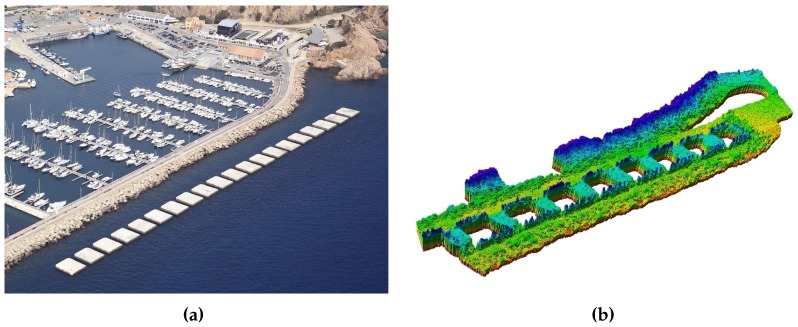
(**a**) breakwater structure in the harbor of Sant Feliu de Guíxols in Catalonia, Spain. The structure is composed of a series of blocks, each of which is 14.5 m long and 12 m wide; (**b**) Octomap created from real-world data obtained with a multibeam sonar.

The *mapping* module incrementally builds a representation of the environment by using data received from different kinds of perception sensors, such as multibeam or mechanically scanned profiling sonars, echosounders, etc. Such sensors provide a range of information about nearby obstacles that, combined with the vehicle’s navigation (position and orientation), permits establishing the free and occupied space with respect to an inertial coordinate frame. In order to represent this data, we use an octree-based framework called Octomap [27], which has three main characteristics that permit efficient modelling such volumetric information. The first characteristic is the probabilistic state representation that not only allows us to modify the map when updated environment information is available, but also protects it from noisy measurements, i.e., a position state considers previous information and calculates its new value according to probabilistic functions. The second characteristic is the capacity of representing unexplored areas, which can be relevant for guiding exploration in unknown environments. Finally, Octomap offers a computationally efficient way to enlarge or extend the map as demanded. Figure 3 shows a breakwater structure and its representation with an Octomap, which has been built using multibeam sonar data obtained by a surface vessel.

### 2.2. Module for (Re)Planning Paths Online

The *planning* module is in charge of calculating a collision-free path for the AUV. For doing so, this module receives a query to be solved that is specified with a start configuration ($q_{start}$) and a goal configuration ($q_{goal}$), and other parameters, such as the available computing time and minimum distance to the goal. Furthermore, given that the vehicle navigates in an unknown environment, this module is required to continuously verify and repair (if necessary) the path from the current vehicle’s position to $q_{goal}$. In order to calculate a collision-free path under such constraints, i.e., incrementally and online, we have modified the asymptotic optimal RRT (RRT*) [28], which is one of the most relevant sampling-based path planning algorithms.

The rapidly-exploring random tree (RRT) and its variants are algorithms that incrementally build a tree of collision-free configurations. Their main characteristic is the rapid and efficient exploration of the C-Space [29]. The state-of-the-art method for calculating optimal paths is the RRT* with its concept of asymptotic optimality (an algorithm is said to be probabilistically complete when the probability that the planner finds a path, if one exists, asymptotically approaches one as the number of samples increases), which was firstly introduced in 2010 by Karaman and Frazzoli [28,30]. This property states that the total cost of the solution, measured by a user-defined function, decreases as the number of samples increases. In this approach, new configurations are connected to the closest and best configuration, i.e., the one that guarantees a minimum cost. Furthermore, an additional step of sample reconnection allows improving the associated cost of the surrounding configurations.

However, the RRT* used in this work not only permits us to progressively improve the path (through its property of asymptotic optimality), but has also been modified to incorporate concepts of *anytime* computation and *delayed collision checking*. These extensions enable the enhancement of the performance for online (re)planning applications, such as the one proposed for this work.

#### 2.2.1. Anytime Approach for (Re)Planning Online

Even though RRT* has been previously extended to behave as an *anytime* algorithm [31], our alternative approach grows a single tree and prunes it to discard those branches that result under collision after updating the map, similarly to what Bekris and Kavraki proposed for a standard RRT [32]. Like other RRT-based algorithms, our variant consists of two procedures, build and extend. The former procedure, which is presented in Algorithm 1, works similarly to other RRTs that sample uniformly distributed configurations (line 4) and attempt to expand the tree towards them (line 5 (see also Algorithm 2)). However, our variant has two main modifications.

The first modification arises from the necessity of correcting or adjusting the path according to the new elements discovered in the environment. In order to deal with this, updateTree procedure is called before sampling new configurations (line 2). With this procedure, the modified RRT* traverses the tree using a depth-first search (DFS) algorithm to check if any node or edge is under collision. If a new collision is detected, the corresponding subtree (i.e., the tree that includes the nodes or edges under collision) will be discarded. Nonetheless, if the tree root is one of the nodes under collision or if the path from the current vehicle’s configuration to the root is not feasible, our modified RRT* (i.e., the *planning module*) informs the *mission handler* to cancel the current waypoint and starts again planning a new path from the current vehicle’s position. This latter situation occurs because the tree root always corresponds to the configuration (or position) that the vehicle is moving towards, as explained below.

The purpose of the second modification in Algorithm 1 is to make the RRT* behave in an *anytime* fashion. To do this, if the new configuration resulted from the tree expansion meets the specified minimum distance to the goal (line 7), it is added to a list of possible solutions (line 8). After concluding the tree expansion, if the *mission handler* has requested a new waypoint and there is at least one available solution stored in the list (line 10), the planner selects the solution with the minimum associated cost, sends the *mission handler* the configuration connected to the root of that solution (line 13), and prunes the tree in such a way that the configuration sent becomes the new tree root (line 14). During this *pruning* process, subtrees connected to the initial root (excepting the corresponding to the new root) are discarded.

In our modified RRT*, extend procedure (Algorithm 2) remains as originally proposed in [28]. This means that it receives a random configuration ($q_{rand}$) towards which the tree will be expanded. To do so, it first finds the node (configuration) $q_{near}$ that is the nearest to $q_{rand}$ (line 2). It then calculates a path of length *δ* from $q_{near}$ towards $q_{rand}$ which, in turn, generates a new configuration $q_{new}$ (line 3) (in a geometrical case, i.e., when no motion constraints are considered, the connection between two configurations results in a straight line segment). If both $q_{new}$ and the path that connects $q_{near}$ and $q_{new}$ are proved to be safe (collision-free), this procedure will not only incorporate $q_{new}$ into the tree, but will also check its surrounding nodes to reconnect them in case better (less expensive) connections are possible (lines 6–9). Finally, this procedure returns a value that confirms whether the expansion was successful (line 10) or not (line 12).

**Algorithm 1:** buildRRT  **Input**:   *T*: tree of collision-free configurations. 
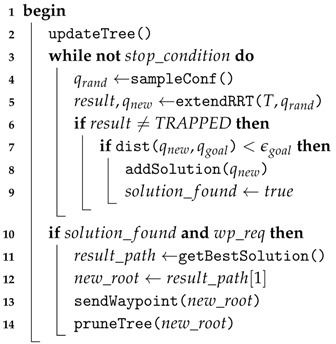


**Algorithm 2:** extendRRT*  **Input**:   *T*: tree of collision-free configurations.  $q_{rand}$: state towards which the tree will be extended.  C: C-Space.  **Output**:   Result after attempting to extend. 
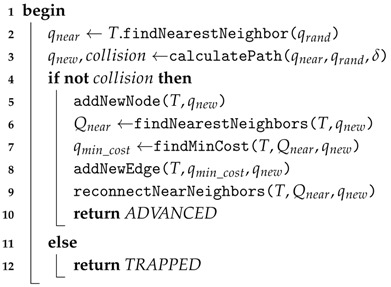


**Figure 4:**
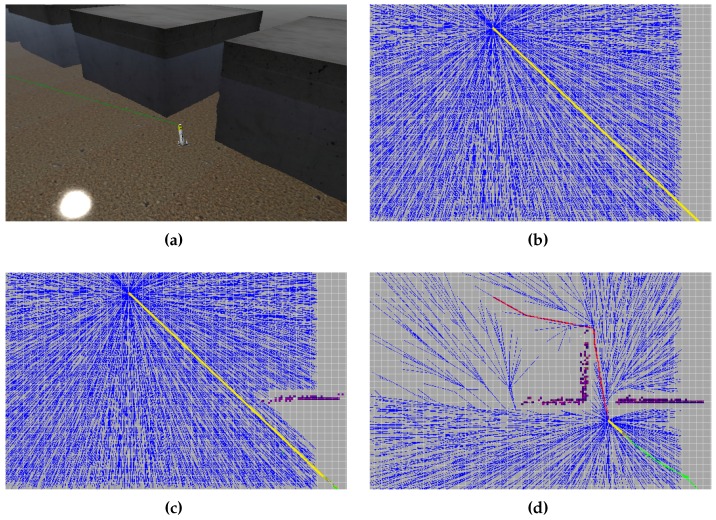
Sparus II AUV conducting an autonomous mission in a simulated scenario (**a**), where it incrementally maps the environment (**b**), (**c**) and (re)plans a collision-free path to the goal (**d**). The tree of configurations is presented in dark blue, the path to the goal in red, the path to the current waypoint in yellow, and the vehicle’s trajectory in green.

Figure 4 shows a simulation of the Sparus II AUV conducting a mission in an environment that resembles the breakwater structure presented in Figure 3. In this case, the vehicle is assumed to have a mechanically scanning profiler with a perception distance of 20 m, the tree generated by our modified RRT* is presented in dark blue, the path to the goal is drawn in red, and the path to the current waypoint appears in yellow. The mission, specified as a start-to-goal query, consists in navigating from one side of a series of blocks (obstacles) to the other (see Figure 4d). The environment is initially undiscovered and is incrementally mapped as the AUV navigates towards the goal. When the vehicle starts the mission, and no obstacle has been detected, the waypoint sent to the AUV’s controllers (the tree root) coincides with the goal, since a straight path to it is feasible (see Figure 4a). This situation can persist even when obstacles have been detected, as long as the path from the vehicle’s position to the goal is collision-free (see Figure 4b). However, when such a straight path is not possible, the planner starts again calculating a new path from the vehicle’s current position, as previously explained (see Figure 4c). More details about the simulation environment and test scenarios will be provided in Section 4.

#### 2.2.2. Delayed Collision Checking for (Re)Planning Incrementally and Online

When navigating in unknown or partially known environments, the information about the static and dynamic surrounding elements (obstacles) is progressively acquired as the vehicle moves. Because of this, an important number of configurations (sampled or obtained after expanding the tree) are located in unexplored regions of the environment. In these situations, it is not only impossible but also unnecessary to attempt to determine if a configuration is at risk of collision. To compensate for this, our strategy is to assume as safe (collision-free) any configuration that is out of the explored area, which can be efficiently determined when using Octomaps (as described in Section 2.1). Furthermore, given that the tree expansion is periodically interleaved with updating the map, such parts initially assumed as safe will be verified and discarded if found under collision as the vehicle explores the environment. This approach is inspired by the *lazy collision checking* strategy introduced by Bohlin and Kavraki [33].

**Figure 5:**
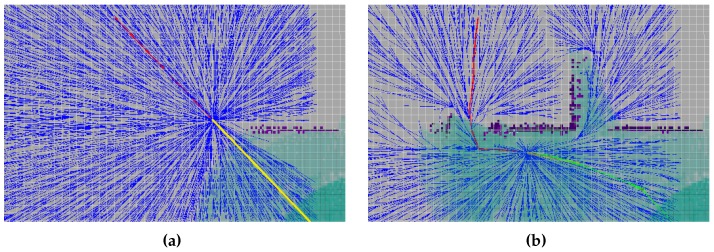
Sparus II AUV conducting an autonomous mission in the same simulated scenario (breakwater-structure). (**a**) The explored region, presented in light blue, expands as the vehicle moves towards the goal. It is important to notice that a significant part of the tree (dark blue) is located in undiscovered areas of the workspace; (**b**) Those branches are initially assumed as safe (collision-free) until the corresponding region has been explored, thus avoiding unnecessary collision-checking routines computation.

Figure 5 depicts another simulation in the same scenario used in Figure 4, but, in this case, the explored regions of the maps are presented in light blue and the occupied ones are presented in green. With this visualization, the importance of delaying the collision checking for those configurations located in undiscovered regions can be appreciated. As mentioned before, the tree is checked and reshaped as the environment is being explored (see changes from Figure 5a–b).

### 2.3. Mission Handler

The third functional module that constitutes the path-planning pipeline is the *mission handler*. This module is in charge of controlling and coordinating the previously explained modules (*mapping* and *planning*). It also verifies whether the AUV is prepared to start solving and conducting a task; to do so, this module communicates with other functional modules on the vehicle to verify both that navigation data is correctly being generated and that the vehicle’s low-level controllers are not conducting any safety manoeuver. After completing the checking stage, the *mission handler* starts requesting waypoints from the *planning* module, which, after being received, are adapted and sent to the vehicle’s low-level controllers. Finally, this module is also responsible for cancelling any ongoing waypoint if it is notified by the *planning* module, as explained in Section 2.2.

### 2.4. Conducting Surveys at a Desired Distance Using a C-Space Costmap

As mentioned in the introduction to this paper, environmental science requires conducting long-term and high-frequency observations in order to determine changes over the studied ecosystem. One of the alternative ways of conducting such a study—and the one presented in this work—is to gather optical data that can be used to build 3D representations of the environment. In this way, changes in the environment can be detected by inspecting the different reconstructions built over time. However, visibility is highly variable in underwater environments and can play a critical role when collecting optical information. For this reason, the planning pipeline has to be adjusted according to both the visibility conditions given when conducting the data collection and the camera parameters.

**Figure 6:**
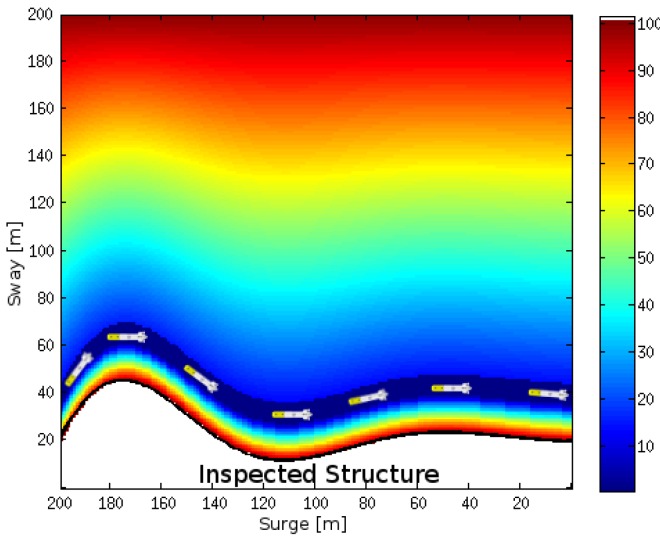
Costmap projected in vehicle’s X(surge)-Y(sway) plane. Dark blue indicates the zone that meets visibility constraints.

In order to tackle this issue, we propose establishing a costmap over the C-Space that specifies a distance constraint as an attempt to guarantee the visibility with respect to the surface of interest. To do so, the cost that is associated with each configuration *q* must be calculated according to Equation (1), where 0≤Cost≤100, with 0 and 100 as the minimum and maximum costs, respectively. This cost function is used as the optimization objective for our planning pipeline and its RRT*. In this formulation, the Cost is dependent on the distance (*d*) and has additional parameters that permit adjusting the costmap. These parameters include the expected distance to the inspected structure ($d_{e}$) and the range of distance ($\Delta d_{a}$) that defines an admissible interval in which the cost is minimal (clearly observed in Figure 6):

(1) $$Cost\left(d\right)=\left\{\begin{array}{cc} \left(1-\frac{d}{d_{e}}\right)100, & d<d_{e}-\frac{\Delta d_{a}}{2},\\ 0, & d_{e}-\frac{\Delta d_{a}}{2}\leq d\leq d_{e}+\frac{\Delta d_{a}}{2},\\ \left(\frac{d}{d_{e}}-1\right)100, & d>d_{e}+\frac{\Delta d_{a}}{2}. \end{array}\right.$$

Lastly, there is an additional important aspect to note when using Equation (1) within the planning pipeline. When high-cost values are defined for certain zones, it does not imply that a restriction is applied to planning paths over those zones (thus discarding possible paths), but it does means that those paths should be avoided as far as possible. To put it in another way, the proposed approach does not define restricted areas in which the vehicle would not be allowed to move through. This situation may occur when the only feasible path coincides with the highest cost one (e.g., narrow passages), in which case the planner will also admit the path as a valid solution.

## 3. 3D Reconstruction Pipeline

Underwater environments with their particular phenomena (i.e., light attenuation, blurring and low contrast) [7], can be regarded as hostile places for optical surveying. Acquisitions have to be performed at close range, significantly limiting the area viewed in a single image, thus enormous amounts of data have to be collected and processed to generate a wide area view enabling the extraction of valuable information on a broader space scale [34]. While 2D photomosaics have been successfully used in describing large areas [13,35,36], they can only be accurately generated if the scene is relatively planar [37] and images taken in a nearly orthographic setup (e.g., [38]). In scenarios with significant 3D structures, the aforementioned prerequisites cannot be met, resulting in obvious distortions (see [39]). However, redundant information from multiple images can be used to provide visually rich 3D reconstructions of the underwater terrain. Furthermore, as the camera poses do not have to be orthogonal to the seafloor, images can convey more meaningful information regarding the global shape of the object, especially in the case of intricate structures (e.g., underwater hydrothermal vents), which are impossible to capture accurately using downward looking cameras [7].

**Figure 7:**
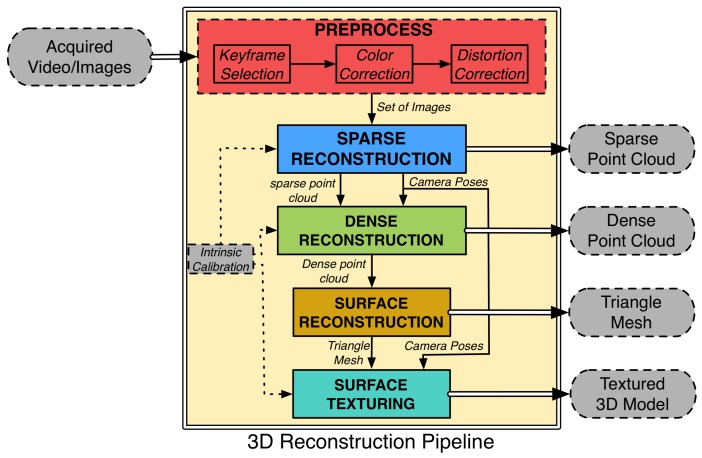
3D reconstruction pipeline.

In this section, an optical-based 3D reconstruction pipeline is presented. Through a series of sequential modules (see Figure 7), the pipeline is able to reconstruct a 3D model based on the optical imagery acquired from an arbitrary number of cameras in various poses. Cameras do not have to be synchronized and can be freely re-positioned between missions, based on the mission’s goals and the expectations of the terrain in the surveyed area. However, in order to obtain a metric-scaled reconstruction, the cameras have to be connected with the AUV sensors [40] or known measurements of the environment have to be introduced [41].

Each of the reconstruction steps results in a different representation of the reconstruction. While the preferable representation for visualization and dissemination is a textured triangle mesh, some applications may require solely dense or even a sparse point cloud of points describing the observed area. In such cases, substantial reduction in the computational cost can be achieved, as none of the subsequent steps have to be performed. It is also worth noting that an important pre-processing step of color correction is required to reduce the image degradation effects of the water medium.

### 3.1. Keyframe Selection

Once the AUV has been recovered, the data acquired during the mission has to be collected and preprocessed in order to obtain a consistent set of quality images used in the subsequent reconstruction process. The optical data can be acquired either by still imagery or through recording of high resolution videos. Challenging underwater conditions in which the acquisition is performed often lead to blurry and low-contrast images, which have to be detected and removed before the start of the reconstruction process. Videos provide a large number of images (i.e., frames) with large overlap and enable a greater flexibility in image selection. However, as the movement of the AUV is typically slow, the length of the videos and consequently the number of frames may become overwhelming and inefficient to process. Furthermore, as the displacement of the camera (i.e., baseline) between consecutive frames is extremely small, the reconstruction process cannot reliably infer the depth information [42]. Thus, a smaller subset of quality images with sufficient movement is extracted and used in the following steps to build an accurate 3D model.

The initial keyframe selection step employed in our reconstruction pipeline is performed as a twofold process on data acquired by each of the cameras used in our setup. To identify the frames in which the vantage point has sufficiently changed, we use a similar approach to Cavan in [43]. The Lucas–Kanade tracking algorithm [44] has been used to track features detected as points with strong gradients in both image directions (minimum eigenvalue corners) [45]. The camera motion is then estimated based on the movement of the tracked features. Despite the fact that this does not allow us to directly estimate real camera movement, it does, however, enable us to detect frames where the content has sufficiently changed. Similarly, if the number of tracked features between the frames is insufficient, we predict a significant change and extract a new keyframe. While Cavan selected each candidate that met this criteria, we perform an additional filtering step to avoid extracting blurry frames. A new frame is selected among a few closely consecutive frames based on the blurriness score, which is estimated as the variance of its Laplacian [35]. As the Laplacian highlights the rapid change of intensities, higher variance represents a more in-focus image with sharper edges.

This approach enables us to automatically adjust the extraction rate of the frames depending on the movement of the AUV, as opposed to time-dependent frame extraction (e.g., selecting a frame every second). However, the approach requires an empirical selection of per-video threshold value for sufficient feature movement on the image plane. On the contrary, the least blurry image is detected based on the relative highest variance and is not conditioned by any thresholds.

### 3.2. Color Correction

**Figure 8:**
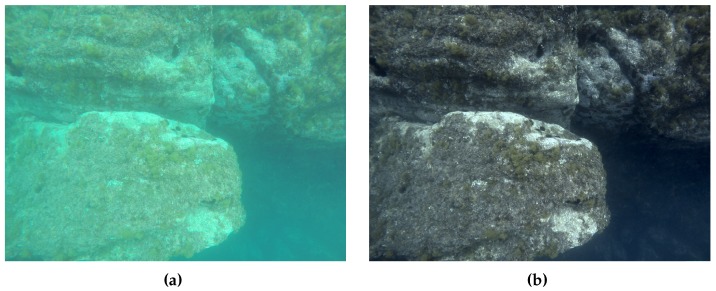
(**a**) image acquired with a downward oriented camera; (**b**) the same image after color correction.

The inherent properties of the water medium induce several effects causing the color and brightness of the images to vary significantly depending on the distance to the scene [46]. The red components of the visible spectrum are strongly absorbed, resulting in typical greenish-blue images [47] (see Figure 8a). The image quality can be further degraded by the scattering and absorption effects caused by the presence of suspended particles in the medium (e.g., plankton) [48,49]. For heavily degraded images, applying color correction not only benefits human perception and interpretation of its contents, but also improves the quality and quantity of successful scale-invariant feature transform (SIFT) matches between image pairs [50] used in the subsequent reconstruction process.

The color correction techniques can be based either on a physical model of the image formulation process (image restoration) or on subjective qualitative criteria (image enhancement). While the former requires knowledge of the physical parameters of the medium, the latter can be performed based on various suggested criteria (e.g., histogram stretching). As our goal is to perform the reconstruction without the requirement of prior knowledge about physical parameters or medium properties, we use an image enhancement technique proposed by Bianco et al. [47]. The method is based on the “white-world” assumption in Ruderman opponent color space lαβ [51] and uniform illumination of the scene. The lαβ space is used to separate the luminance (*l*) and two chromatic components (α,β) and subsequently shift their distributions around the white point (0,0). This can be seen as the correction of image-cast and the adjustment is analogous to the “grey-world” assumption in the RGB space. Additionally, histogram cutoff and stretching are performed on the luminance component to improve the image contrast. An example image before and after color correction and contrast enhancement is depicted in Figure 8.

### 3.3. Distortion Correction

Aside from the effects of the light passing through water, the incident light beam’s path is additionally altered due to the difference in the density in the water–glass–air interface (between the camera sensor and the scene). The change in path destroys the collinearity between the point in water, the camera’s center of projection, and the image point [52], resulting in a distorted image, causing the scene to appear wider on the image than it actually is [53].

For planar interfaces, such as we use (see Section 4.1), the deformation increases with respect to the distance from the camera’s center of projection. It can be regarded as a pin-cushion distortion, and its effects can be reduced by considering it as radial distortion [54]. Unknown radial distortion parameters introduce additional ambiguity in the subsequent structure from motion (SfM) process and can lead to ambiguous reconstructions (e.g., bending of the model) [55]. This can be avoided by either using pre-calibrated radial distortion parameters or avoiding critical acquisition configurations [55].

As we cannot ensure the absence of critical configurations, due to the unknown structure of the environment, we pre-calibrate the intrinsic parameters of the cameras in an underwater environment prior to the mission using a calibration pattern and a standard calibration procedure [56].

### 3.4. Sparse Reconstruction

Using a set of previously pre-processed images, the 3D geometry of the scene, simplified to sparse 3D points, is simultaneously estimated with the motion of the cameras through a process known as structure from motion (SfM). The problem solved by SfM can be seen as an inverse process of image formulation. Instead of finding the points of intersection between the image plane and the rays connecting the camera’s centre of projection and the points in space, the goal is to recover the position of the points in space together with the pose of the camera. The estimation is done entirely from the texture features extracted and matched across the 2D image set. By using the equations of projective geometry, connecting the image projections and the position of real world points, the solution is estimated through a non-linear minimization of the reprojection errors—also known as bundle adjustment [57]. As the process is based on projective geometry, the obtained reconstruction can be defined only up to scale [42].

#### 3.4.1. Feature Detection and Matching

Given that the structure and motion parameters are inferred entirely from the projections of the points on the images, these interest points should be salient features robustly detected and associated across multiple views. In our approach, we detect such features using Wu’s [58] graphics processing unit (GPU) implementation of SIFT [59]. SIFT is widely accepted as one of the highest quality feature descriptors [60] as it has a high degree of invariance to scale and rotation, as well as being partially invariant to changes in illumination, noise, occlusions and small changes in the viewpoint. The points are detected as extremes of the difference of gaussians (DOG) at multiple scales, and described based on local gradients using a 128-element normalized unit vector.

The association of features across the image set is done image pairwise based on the Euclidean distance using Lowe’s ratio test [59] and subsequently filtered through a geometric filtering procedure to eliminate possible outliers. The behaviour of individual matches is evaluated with respect to the global estimate of the transformation between the two images, i.e., epipolar constraints [42]. Without prior knowledge of the cameras positions, the fundamental/essential matrices are computed using a robust statistical method to prevent the influence of possible outliers on the estimation of the model. In order to avoid the empirical selection of the inlier/outlier threshold value in the widely used method of random sample consensus (RANSAC) [61], we use the parameter-free evolution called a contrario-RANSAC (AC-RANSAC) [62] implemented in the open-source library OpenMVG [63]. The method uses the *acontrario* methodology, which relies on the Helmholtz principle of meaningful deviations and regards any model that is unlikely to be explained by chance as conspicuous. As the meaningfulness of the model is determined by data-specific statistical criteria, explicit threshold values are not required.

#### 3.4.2. Structure from Motion

Using the established feature correspondences, the 3D scene and extrinsic camera parameters are gradually estimated through a sequential SfM [64] implemented in the open-source library OpenMVG [63]. The process starts with the initialization step, where a seed pair of images is selected. Without prior knowledge of the scene, initial poses are decomposed directly from the estimated fundamental/essential matrix relating the pair. As the erroneous initial estimation can cause the algorithm to converge to wrong local minima, from which it is unlikely to recover, the selected pair has to be well conditioned, i.e., have a wide baseline and not many coplanar common points, to ensure robust estimation of the fundamental/essential matrix [42]. The reconstruction is then incrementally expanded with newly observed points and cameras one view at a time. Extrinsic parameters of each new camera are initialized through a direct linear transform technique [42] using already estimated 3D points. After each step of the expansion, bundle adjustment is performed to minimize and evenly propagate the re-projection error.

As the reconstruction is performed sequentially from a series of relative motion estimations between the images, the accumulation of small errors can lead to a drift in the final reconstruction. This can be significantly reduced with the introduction of non-sequential constraints in the optimization process. An effective strategy is to perform a loop closure by re-visiting the same area. Using the image matching between the non-sequential images the relative motion is restricted and drift minimized.

While the algorithm enables the recovery of both extrinsic (i.e., the position and orientation of the camera at the moment of the acquisition) and intrinsic cameras parameters (i.e., focal length, principle points and radial distortions of the lens), this can lead to ambiguous reconstructions (excluding the inherited scale ambiguity) [55] (see Section 3.3). We avoid the radial ambiguity by pre-calibrating the cameras in an underwater environment and consider the intrinsic parameters constant during the reconstruction process. This additionally reduces the complexity of the problem and subsequently reduces the possibility of convergence to a wrong solution.

**Figure 9:**
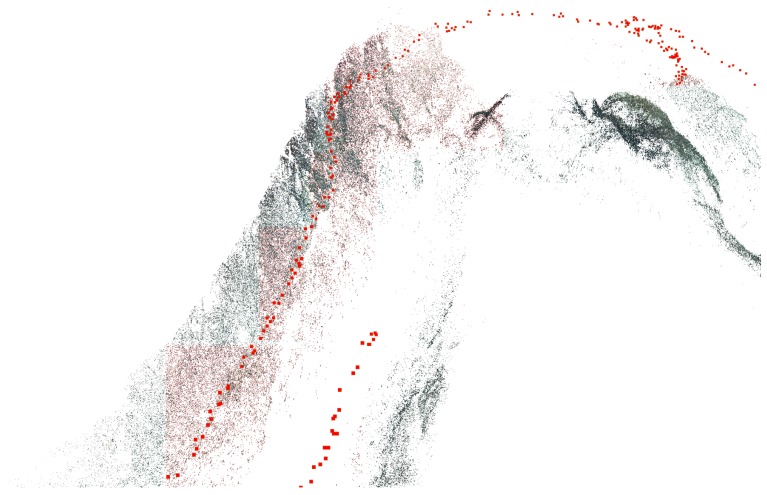
Example of sparse 3D scene reconstruction together with with camera positions (red).

As a final result, an estimate of the external camera parameters together with the sparse 3D structure is obtained as shown in Figure 9.

### 3.5. Dense Reconstruction

As sparse 3D points describing the observed scene recovered in the previous step are usually not sufficient to describe the underlining structure in detail, we perform a densification step in which we aim to obtain a detailed globally consistent dense representation of the scene. This is achieved by trying to estimate the 3D coordinates of each pixel in the acquired images. Using the mathematical models estimated in the SfM process (e.g., camera projection matrices) [65], the correspondence information required for such estimations can be determined through a dense image matching procedure, either by using stereo pairs (stereo matching) or by identifying correspondences in multiple images (multi-view stereo).

Modern dense matching algorithms can be categorized either as local or global [66]. While local methods are efficient, due to pixel-wise correspondence evaluation and local “winner-take-all” optimization [65], they tend to produce incorrect results at sudden depth variations and detailed areas, as well as lacking the ability to reconstruct surfaces in locally ambiguous areas (e.g., occlusions, repeated patterns and uniform texture regions) [67]. Relative robustness in such areas can be achieved using global methods, which determine the solution by minimizing a global cost function extended to all image pixels [68] but require significant computational effort. An efficient solution can be found using a Semi-Global Matching [69] method, which is a local pixel-wise method that approximates the minimization of a global 2D smoothness constraint by combining several independent 1D constraints, thus allowing the recovery of object boundaries and fine details.

We perform the dense reconstruction using a semi-global matching-like multi-image method [70,71] implemented in an open-source MicMac photogrammetry [72]. The reconstruction is formulated as an energy minimization problem and solved by finding a minimal cut in a graph. For each hypothetical 3D point, a patch in the master image is identified and projected to all the neighbouring images, which are then used in the computation of the global similarity estimate through Normalized Cross Correlation [65]. An energy minimization approach, similar to [69], is then applied to enforce surface regularities and avoid undesirable jumps [71].

**Figure 10:**
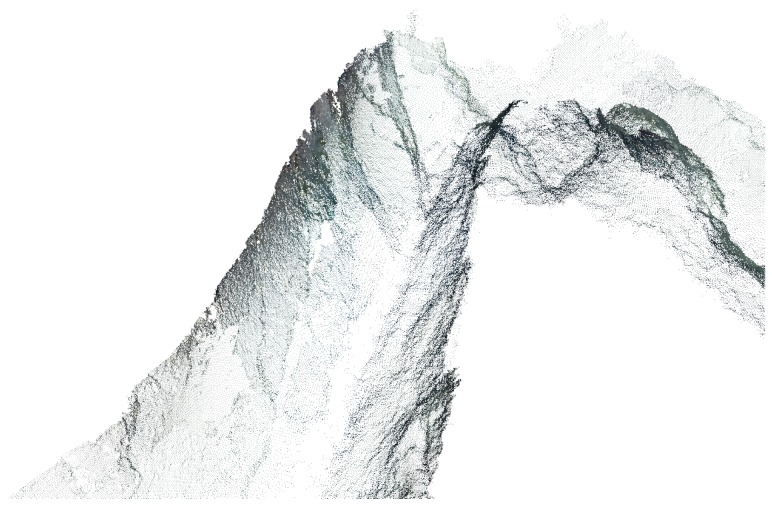
Example of dense 3D scene reconstruction.

To reduce the computational complexity, the method is based on a multi-resolution pyramidal approach using a coarse-to-fine extension of the maximum-flow image matching algorithm presented in [73]. At each pyramid level, matching results for the relevant resolution are computed and used to guide the matching process at a higher level. This produces a multi-stereo correlation result for each master image in the form of a depth map. A 3D point cloud is later obtained by projecting the points to space according to the camera’s pose and associated depth value. Additionally, RGB attributes can be assigned to each of the 3D points from the appropriate master image [71]. The result can be seen in Figure 10, depicting the scene from the same viewpoint as in Figure 9.

### 3.6. Surface Reconstruction

In sparse and dense point clouds obtained in the previous steps, the scene has only been described using an unorganized noisy point cloud without any assumption about their connectivity. This representation is frequently not sufficient for further processing of the data, and it also does not enable proper visualization, as visibility information is not established, preventing the user from easily distinguishing points that should be either visible or occluded from a specific viewpoint [7].

In the surface reconstruction step, our aim is to describe the geometry of the scene using a triangle mesh, i.e., finding the most probable surface based on the sampling represented by the point cloud obtained from the noisy dense reconstruction. Optical-based reconstructions created in the underwater environment are normally corrupted by both noise and outliers due to poor imaging conditions [7]. Surface reconstruction methods can be classified as interpolation- or approximation-based depending on their approach [7]. Interpolation methods consider the points in the point set only as possible vertex candidates in the resulting triangle mesh, thus making them directly dependent on the quality of the point cloud. For this reason, such methods should only be used with ideal (or nearly ideal) point clouds, i.e., noise and outlier free [7]. On the contrary, the approximation-based methods are able to mitigate the effects of noise in the data by considering the point set only as information about the surface and not necessarily as final vertices. However, the implicit smoothing hampers its ability to recover sharp features (e.g., edges, corners). Considering that our main focus is on the recovery of the scenes in the underwater scenarios, where sharp edges are rare (with the exception of man-made structures) and the point cloud is noisy, we decided to use the Poisson method [74,75] as one of the most representative approximation-based methods.

The Poisson surface reconstruction method forms a unique implicit surface representation (iso-surface) through the reconstruction of an indicator function, i.e., a function having the value 0 if inside the object, and 1 if outside. As the oriented points in the point cloud can be seen as samples of the indicator function’s gradient, the point cloud can be used to represent the function’s gradient field. By computing the inverse of the gradient, that is, by finding the scalar function of which gradient best approximates the gradient field defined by the input point set, the problem can be transformed to a Poisson problem and its solution found by determining the scalar function whose Laplacian equals the divergence of the gradient field. To efficiently represent the 3D function, an octree adapted to the distribution of the samples is used.

**Figure 11:**
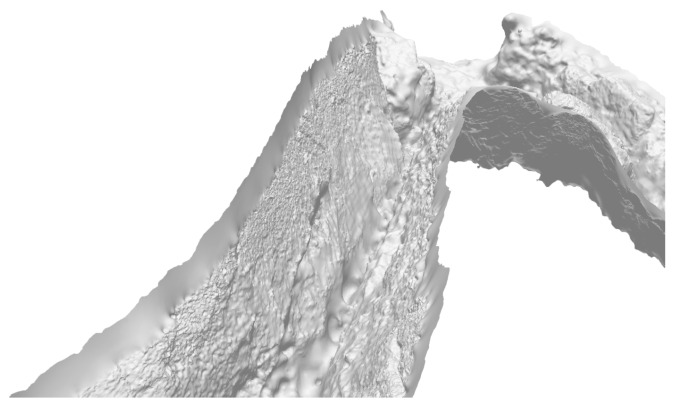
Example of surface reconstruction.

By using the indicator function, the Poisson method implicitly requires the possibility of determining the inside and outside of the surface, i.e., the object has to be watertight [7]. As the underwater scenarios are predominately focused on the reconstruction of the seafloor with various additional 3D structures, the scene can not be properly viewed from all angles. Such scenarios force the Poisson method to define false surfaces in areas lacking needed information. These areas can be subsequently eliminated by removing triangles with edges longer than a certain threshold (triangles increase in size in non-sampled parts) [76]. The resulting model is presented in Figure 11.

### 3.7. Surface Texturing

In the final step of our reconstruction pipeline, the goal is to obtain a photo-realistic 3D model of the observed scene. A consistent texture for the reconstructed surface mesh can be retrieved by mapping high-quality textures from the input images.

Generating a globally consistent and seamless texture is challenging due to changes in the acquisition conditions (e.g., changes in illumination, light attenuation, white-balancing, presence of unreconstructed occluding objects (particles in the water)), varying image scales (e.g., close-ups, distant overview images), as well as unavoidable imperfections in the reconstructed geometry) [77]. As multiple images observe each surface element, their information can either be fused using blending techniques or the information from a selected image can be used. In blending, due to the inaccuracies in the camera poses, slight inaccuracies in the reconstruction and different attenuation of the light depending on the camera poses, can lead to ghosting and blurring of details in the final texture. Alternatively, by selecting the most appropriate image for each texel, seams between regions mapped from different images can be made clearly visible [78].

In our pipeline, we use the work of Waechter et al. [77], which performs the mapping of textures from multiple registered images in two steps. First, for each surface face, a single image is selected through a process of energy minimization, preferring close focused orthogonal views with high resolution and similar adjacent patches. Additional photo-consistency checking is employed to detect and reject any inconsistent views caused by unreconstructed occluding objects. Color discontinuities between the patches are then adjusted in an attempt to minimize the visibility of the seams. Per-vertex-based globally optimal luminance correction terms are computed using a weighted average of the vertex color along all adjacent seam edges, and are used together with local Poisson image editing [79] to generate the final coherent texture of the reconstructed scene.

**Figure 12:**
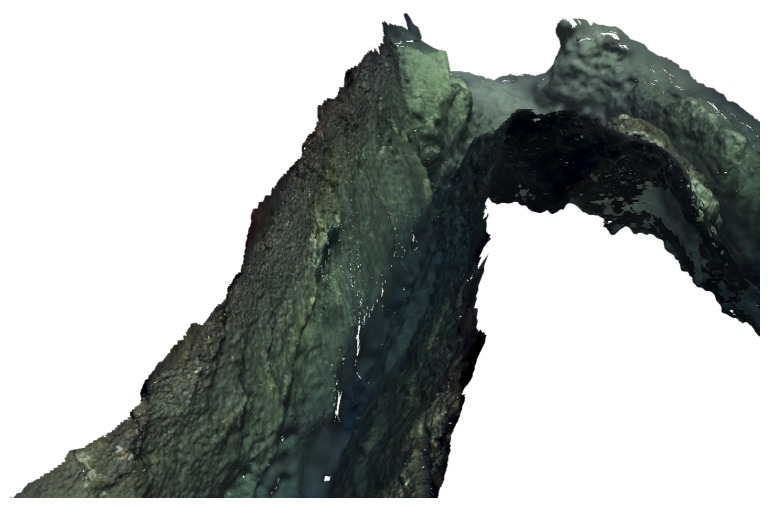
Example of surface reconstruction.

An arbitrary user-defined view of the textured 3D model is presented in Figure 12.

## 4. Results

In order to evaluate and validate the presented approach, we selected a natural and unexplored underwater scenario in which the Sparus II AUV had to autonomously navigate. The vehicle not only successfully conducted multiple start-to-goal missions, but also gathered optical data that was used to build a 3D reconstruction of the surroundings. The results demonstrate the capabilities of our approach in natural real-world conditions, and validate our preliminary work conducted in a simulated environment [26]. This section explains the vehicle setup and presents the results obtained in one of the conducted missions.

### 4.1. Experimental Setup and Simulation Environment

**Figure 13:**
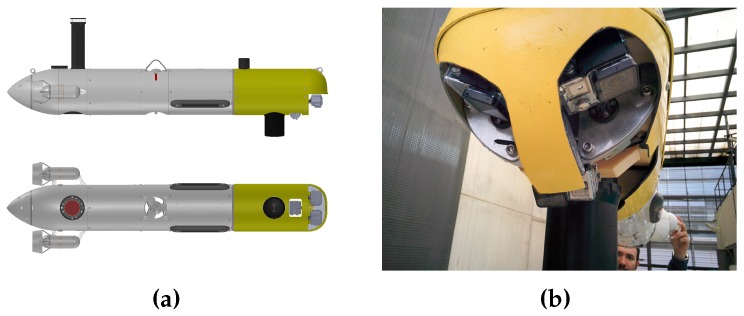
Sparus II AUV. (**a**) CAD model, where the three thrusters can be observed, as well as the profiling sonar and cameras located in the payload area (yellow); (**b**) real-world vehicle’s payload.

Sparus II is the most recent AUV developed at the CIRS [80]. Rated for depths up to 200 m, the torpedo-shaped robot has three thrusters (two horizontal and one vertical, see Figure 13a) and can be actuated in surge, heave and yaw modes degrees of freedom (DOF), which endows it with hovering capabilities. For estimating its position and orientation, the vehicle is equipped with a navigation sensor suite that includes a pressure sensor, a doppler velocity log (DVL), an inertial measurement unit (IMU) and a GPS to receive fixes while at surface. Furthermore, the vehicle has different perception sensors that are located within the vehicle’s payload (front) area, including a mechanically scanning pencil-beam sonar used online to create the surroundings map, and a set of three GoPro Hero 4 Black edition cameras (GoPro, San Mateo, CA, United States) that gather the images required to create the 3D environment’s reconstruction. The cameras are positioned systematically (see Figure 13) to ensure the highest possible coverage, while still maintaining the ability to perform feature matching between images taken from different perspectives. Two cameras were placed in a downward configuration at an angle of 20 degrees while the third camera was positioned forward looking at 40 degrees.

**Figure 14:**
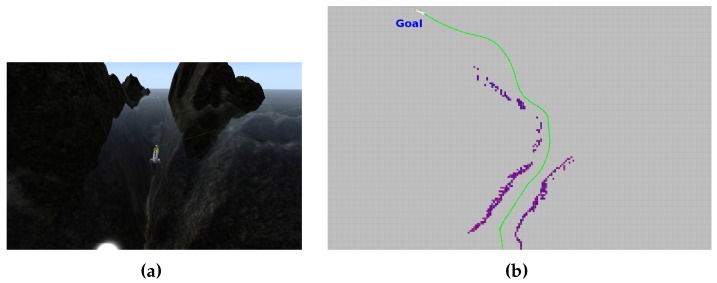
(**a**) Sparus II AUV conducting autonomous missions in a simulated environment (UWSim), which resembles an underwater canyron created by a rocky formation; (**b**) The vehicle after traveled successfully through the canyon. The map, generated online, can be observed in purple, while the vehicle trajectory appears in green.

Sparus II AUV is controlled through the component oriented layer-based architecture for autonomy (COLA2) [81], which is completely integrated with the robot operating system (ROS). Furthermore, COLA2 not only operates the real vehicle, but can also interact with the underwater simulator (UWSim) [82], thus permitting the use of 3D environment models and simulation of the vehicle’s sensors and dynamics. Before conducting real-world trials, we used UWSim with different virtual scenarios in order to extensively simulate and test most of the vehicle’s functional modules, including our path-planning pipeline. For this particular work, we designed two virtual scenarios: one that resembles the breakwater structure mentioned in Section 2 (see Figure 3 and Figure 4), and one that includes an underwater canyon located between two rocks (see Figure 14). In both cases, the vehicle succeeded in conducting start-to-goal missions without having a priori information on the environment. Another important aspect to mention is that we make use of the open motion planning library (OMPL), which is a general path-planning library that can be extended and adapted to different contexts and problems [83].

The following sections present an inspection mission conducted by the Sparus II AUV in a real-world and natural environment. The results include not only the navigation map created online from the profiling sonar data, but also the 3D reconstruction of the underwater surroundings.

### 4.2. Online Mapping and Path Planning in Unexplored Natural Environments

**Figure 15:**
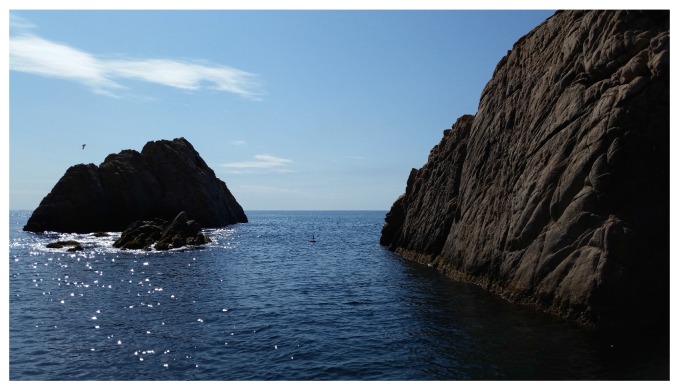
The test scenario that consists of rocky formations that create an underwater canyon.

In order to evaluate our approach, we tested its effectiveness in a challenging real-world natural environment in Sant Feliu de Guíxols, Spain (see Figure 15). The testing area contains rocky formations that create an underwater canyon. In order to inspect this environment, two different start-to-goal queries were established by extracting GPS coordinates from Google Maps [84]. The first query required the Sparus II AUV to traverse the canyon towards the shore. The second query goal was chosen on the outside of the rocky formation in such a way that the vehicle had to circumnavigate the outer rock. Furthermore, after completing the second query, the first query was executed again until the vehicle overlapped its initial trajectory in the canyon, in order to close the imaging acquisition loop and thus improve the reconstruction results. The profiling sonar of the AUV only covers the horizontal plane, which restricts the safe motion of the vehicle to planes of constant depth. For this reason, the navigation was set at a constant depth of 3 m for both queries.

**Figure 16:**
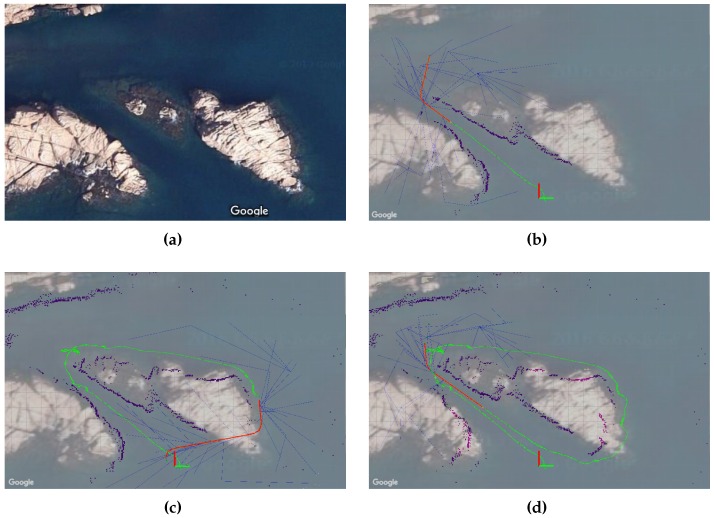
(**a**) the test scenario consists of rocky formations that create an underwater canyon; (**b**) Sparus II AUV conducting the first part of the inspection mission that requires traversing the canyon; (**c**) during the second part of the mission, the vehicle circumnavigates one of the rocks on its way back to the initial position; (**d**) the AUV partially repeats the first start-to-goal query in order to close the loop and obtain overlapped images.

Figure 16 depicts the results of the inspection mission, where the AUV not only created a map of a complex and unknown environment, but also planned a collision-free path, simultaneously and incrementally. The map and the vehicle’s trajectory are shown overlapping a satellite image. In the initial part of the mission, i.e., when the vehicle traverses the canyon for the first time, the map coincides with the satellite image (see Figure 16b); however, disparities can be clearly observed after some time (see Figure 16c,d). Such differences are due to the accumulation of errors in the dead reckoning system (position and orientation estimation) that depends on the DVL, which may provide incorrect data when navigating over rocks, as occurred in this test scenario. Despite this situation, the vehicle succeeded in conducting the mission because both the map and the path are created online, which permits correcting or adjusting them even when moving in previously visited areas (see Figure 16d when accessing the canyon for a second time).

### 4.3. 3D Reconstruction

During the autonomous inspection mission, each of the three cameras captured 725 seconds of video, comprising a total of 21,750 frames. The imagery was acquired in 2.7 K HD 4:3 video mode with a resolution of 2704×2028 pixels. In order to test our pipeline, we used the complete set of frames as input. Using the keyframe selection method described in Section 3.1, we automatically identified 264, 376, and 316 representative frames from the left, right and forward looking camera, respectively.

**Figure 17:**
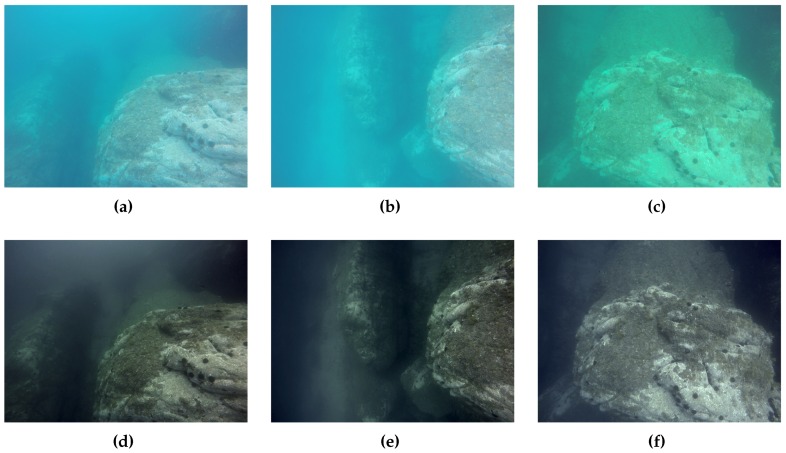
(**a**–**c**) images captured by forward, left and right camera respectively; (**d**–**f**) images after color correction and contrast enhancement.

The visibility varied between different sections of the mission from ∼4.5 m inside the canyon to ∼3 m on the most exposed outer part. These conditions, combined with the different viewing angles of the cameras, caused the acquired images to have low contrast and different appearances depending on the cameras, with predominant blue and green tones (see Figure 17a–c). This was especially noticeable in the last part of the mission, where the AUV pulled away from the rocky formation. For this reason, the images were color corrected and contrast enhanced, to unify the appearance and improve the feature detection and matching (see Figure 17d–f).

**Figure 18:**
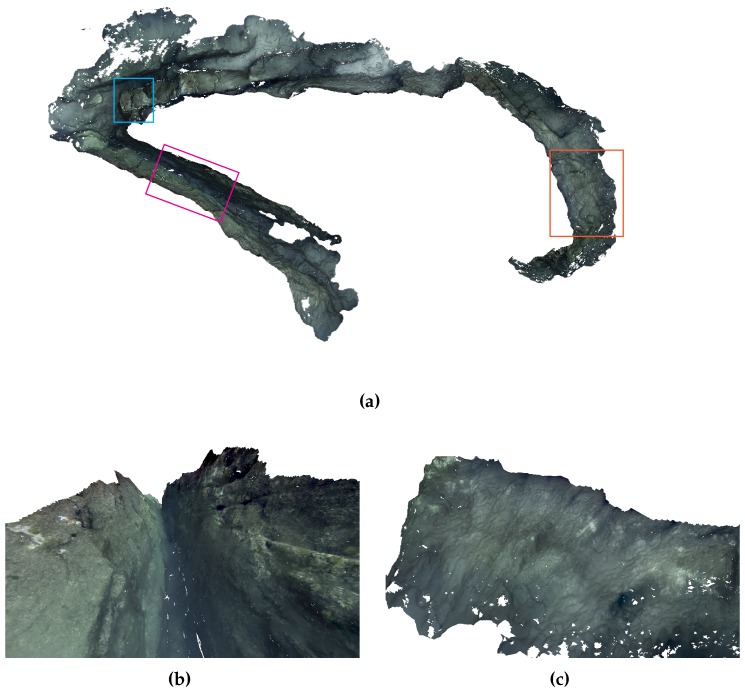
(**a**) top-down view of the textured 3D model with marked areas additionally depicted (magenta–Figure 18b, orange–Figure 18b and blue–Figure 19b); (**b**) generated view inside the underwater canyon; (**c**) generated view of the external side of the underwater rocky formation.

**Figure 19:**
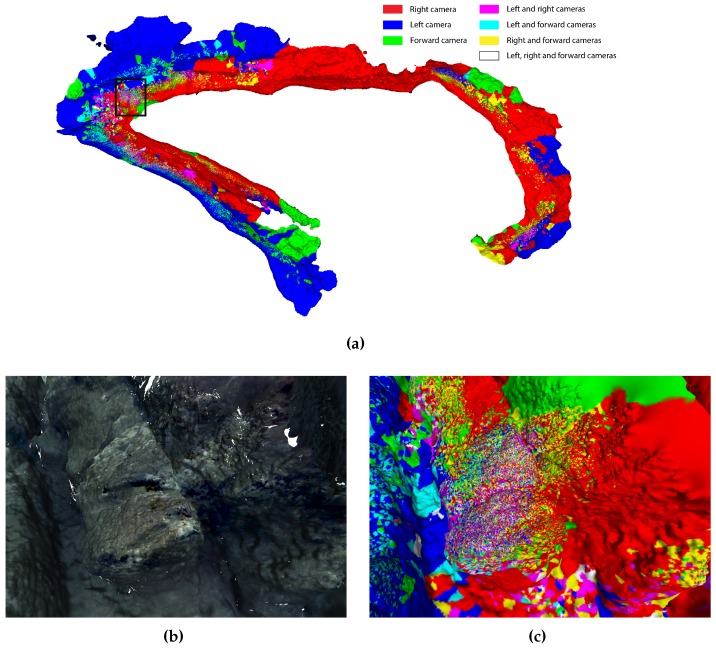
(**a**) color coded 3D reconstruction based on the cameras used in specific areas in top-down view; (**b**) generated view of a marked area in blue in Figure 19a using textured 3D model; (**c**) color coded view of the same view as Figure 19b.

The reconstruction was obtained using the series of sequential steps explained in Section 3, each producing an intermediate representation, e.g., sparse/dense point clouds and 3D triangle mesh (see Figure 9, Figure 10 and Figure 11), as well as the final photo-realistic 3D model depicted in Figure 18a, presented in a standard top-down view. The recovered 3D model also enables us to generate arbitrary user-defined views (see Figure 18b,c and Figure 19b, and also supplementary material Video S1 to observe the complete reconstruction). Since the reconstruction is performed exclusively from image information, the areas which were not properly imaged (such as crevices, or areas occluded by other rocks along the robot path) can not be reconstructed. Similarly, the bottom of the canyon was not properly observed on the images due to the poor visibility conditions, thus preventing its reconstruction in high detail.

While a 3D reconstruction could be achieved with a single camera, each of the cameras used in our mission contributed an important part of the final result. This can be seen in Figure 19a where the reconstruction is color coded with respect to the different combinations of cameras used for different areas. It is not surprising that the largest area is reconstructed using the images captured by the right camera, as this camera was within the visibility range of the rocky formations. This was not true for the remaining cameras, as the visibility conditions on several occasions prevented the observation of any distant objects. While the majority of the reconstruction is done using the combination of one or two cameras (either due to the visibility conditions, or small overlap between downward oriented cameras), we can see that the area in which the texture is especially rich (enlarged area in Figure 19b), many points have been reconstructed from different combinations of cameras (see Figure 19c). When features detected are sufficiently dominant, they can be successfully matched across various views from different cameras.

**Figure 20:**
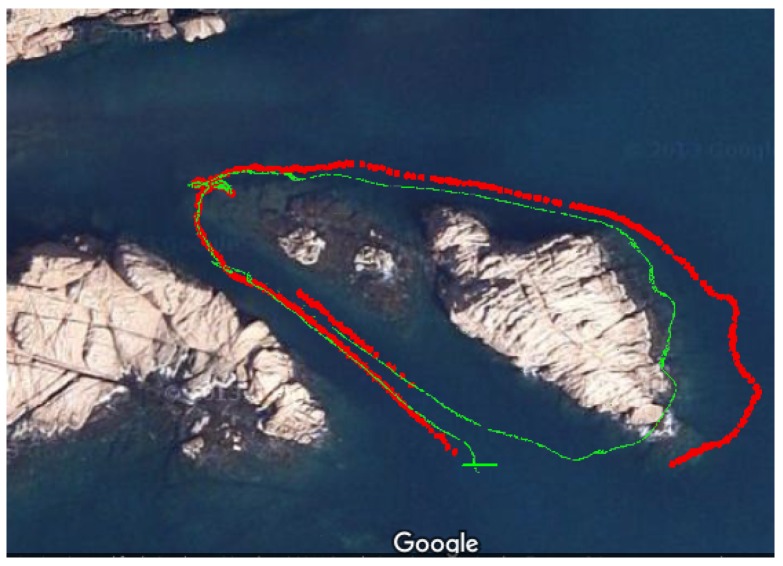
Vehicle’s trayectory (green) calculated by its dead reckoning system and the camera’s trajectory (red) estimated by the SfM, both overlapping a satellite image of the test scenario.

Since the cameras are not connected or synchronized with the AUV, additional information from other sensors cannot be directly imposed in the reconstruction process. As a consequence, the problem solved by the SfM does not allow determination of the real scale of the reconstruction. In order to compare the vehicles and cameras trajectory estimated during the SfM, we manually estimated the scale of the reconstruction. Despite this, Figure 20 presents a visual comparison between them (green for the vehicle and red for the cameras). While both trajectories have a similar shape, it can be clearly observed how the one derived from the cameras is more realistic according to the rock observed in the surface (the rocky formation does not create a vertical wall, which means that the vehicle may have moved further from the visible part of the rock when navigating at 3 m deep), while the one estimated by the AUV’s dead reckoning system seems to be colliding with the rock. This latter situation, as explained the previous section, is mainly due to the accumulation of errors in the navigation system.

A final and important observation in Figure 20 is the loss of visibility in the last part of the mission. Even though the path-planning pipeline attempts to guarantee the visibility of the inspected structure, the second query goal guided the vehicle towards the coordinate system origin (see Figure 16c), which clearly moved the vehicle away from the structure. However, even though such (visibility) discontinuity can be appreciated in the cameras’ trajectory, it can also be observed how the reconstruction pipeline was able to properly match the subsequent images once the vehicle approached the canyon during the second travel. While in this particular case this prevented us from successfully enforcing the loop closure, it does, however, demonstrate the pipeline’s ability if the visibility conditions are met.

## 5. Conclusions

In this paper, we have presented a new end-to-end approach for autonomously mapping unknown underwater natural environments using AUV. To do so, we proposed a framework composed of two main functional pipelines. The first provides the AUV with the capability for creating an acoustic map online, while simultaneously planning collision-free paths. Such functionality is essential for safe navigation in unknown and potentially dangerous areas, and to maintain very short distance to the areas being imaged, as required by optical mapping underwater. Using the gathered image data, the second pipeline builds a photo-realistic 3D model, which can be used as base maps for environmental inspection and subsequent monitoring.

In recent previous work, both pipelines were independently tested in simulated and real-world non-natural (structured) environments. In order to thoroughly validate our approach, this paper presented results obtained in a challenging real-world natural scenario, in which the Sparus II AUV conducted several autonomous missions. The test scenario, containing an underwater canyon between two rocky formations, permitted us to demonstrate the extent of the capabilities of our approach. By successfully navigating through the natural environment, the AUV was able to acquire data subsequently used in the reconstruction of a complex textured 3D model of the area.

Our next effort will focus on using different perception modalities (such as multibeam sonar) to create online acoustic 3D maps, thus enabling missions at different depths. This will permit the path-planning pipeline to attempt maintaining the visibility not only with the inspected structure but also with the sea bottom if desired. Consequently, these kind of missions will allow for better exploitation of the reconstruction pipeline capabilities for representing complex 3D environments.

## Supplementary Materials

The following are available online at https://www.youtube.com/watch?v=ide5gj6V0GM&feature=youtu.be. Video S1: 3D reconstruction created using optical data acquired by an SPARUS II AUV during an autonomous mission in an unknown environment as presented in the paper.

## Abbreviations

The following abbreviations are used in this manuscript:

C-Space: configuration space
RRT: rapidly-exploring random tree
RRT*: asymptotic optimal RRT
1D: 1-dimensional
2D: 2-dimensional
3D: 3-dimensional
OMPL: open motion planning library
DFS: depth-first search
DOF: degrees of freedom
ROS: robot operating system
UUV: unmanned underwater vehicle
ROV: remotely operated vehicle
AUV: autonomous underwater vehicle
DVL: Doppler velocity log
IMU: inertial measurement unit
CIRS: underwater vision and robotics research center
COLA2: component oriented layer-based architecture for autonomy
UWSim: underwater simulator
SIFT: scale-invariant feature transform
SfM: structure from motion
DOG: difference of Gaussians
RANSAC: random sample consensus
AC-RANSAC: a contrario-RANSAC
GPU: graphics processing unit
CAD: computer-aided design
